# Reduced VDAC1, Maintained Mitochondrial Dynamics and Enhanced Mitochondrial Biogenesis in a Transgenic Tau Mouse Model of Alzheimer’s Disease

**DOI:** 10.3390/ijms23158561

**Published:** 2022-08-02

**Authors:** Murali Vijayan, P. Hemachandra Reddy

**Affiliations:** 1 Department of Internal Medicine, Texas Tech University Health Sciences Center, Lubbock, TX 79430, USA; 2 Department of Pharmacology and Neuroscience, Texas Tech University Health Sciences Center, Lubbock, TX 79430, USA; 3 Department of Neurology, Texas Tech University Health Sciences Center, Lubbock, TX 79430, USA; 4 Department of Public Health, Graduate School of Biomedical Sciences, Texas Tech University Health Sciences Center, Lubbock, TX 79430, USA; 5 Department of Speech, Language, and Hearing Sciences, Texas Tech University Health Sciences Center, Lubbock, TX 79430, USA; 6 Nutritional Sciences Department, College of Human Sciences, Texas Tech University, 1301 Akron Ave, Lubbock, TX 79409, USA

**Keywords:** voltage-dependent anion channel 1, mitochondria, fission and fusion, biogenesis, Alzheimer’s disease

## Abstract

Alzheimer’s disease (AD) is one of the most common forms of neurodegeneration, defined by reduced cognitive function, which is caused by the gradual death of neurons in the brain. Recent studies have shown an age-dependent rise in the levels of voltage-dependent anion channel 1 (VDAC1) in AD. In addition, we discovered an aberrant interaction between VDAC1 and P-TAU in the brains of AD patients, which led to abnormalities in the structural and functional integrity of the mitochondria. The purpose of our study is to understand the protective effects of reduced VDAC1 against impaired mitochondrial dynamics and defective mitochondrial biogenesis in transgenic TAU mice. Recently, we crossed heterozygote VDAC1 knockout (VDAC1^+/−^) mice with transgenic TAU mice to obtain double-mutant VDAC1^+/−^/TAU mice. Our goal was to evaluate whether a partial decrease in VDAC1 lessens the amount of mitochondrial toxicity in transgenic Tau (P301L) mice. We found that mitochondrial fission proteins were significantly reduced, and mitochondrial fusion and biogenesis proteins were increased in double-mutant mice compared to TAU mice. On the basis of these discoveries, the current work may have significance for the development of reduced-VDAC1-based treatments for individuals suffering from AD as well as other tauopathies.

## 1. Introduction

Alzheimer’s disease (AD) is the most prevalent neurodegenerative illness. AD is defined by a gradual deterioration in cognitive function, and it is the largest risk factor for developing Alzheimer’s dementia in aged individuals [1]. Before the beginning of clinical symptoms and neuronal death, the illness is characterized by the buildup of amyloid-β (Aβ) peptides and the aggregation of hyperphosphorylated TAU protein (P-TAU), both of which lead to increased mitochondrial dysfunction and synaptic damage [2,3]. There has been a rise in both the prevalence and incidence of AD throughout the world. It is estimated that by the year 2050, 1 in 85 people across the globe will be living with the disease and that 43 percent of those who are affected will require high levels of care [4]. Several decades of intense research revealed that both modifiable and non-modifiable factors have a role in the development of AD [5]. Although tremendous progress has been made in understanding the biology of AD, the fundamental mechanism that drives the pathogenesis of AD is still a mystery.

Mitochondria are important organelles that are present in mammalian cells and are renowned for their crucial function in energy metabolism and cell survival [6]. Mitochondrial biogenesis is an important process that occurs throughout the life cycle of mitochondria. Due to the tremendous dependency of neurons on their metabolic rates and the complexity of their morphologies, neurons are especially susceptible to mitochondrial malfunction [7]. To be more precise, mitochondria play a critical role in the process of synapse formation and plasticity. Notably, abnormalities in mitochondrial activity constitute a key early occurrence in AD [8,9]. New data reveal that mitochondria are dynamic organelles that are constantly undergoing fission and fusion, which is governed by a mechanism that involves highly conserved dynamin-related GTPases [10]. Given the essential function that mitochondria play in neurons, disturbances in the dynamics of mitochondria are increasingly being linked to other neurodegenerative conditions [7,11,12,13].

The inner membrane surrounding the mitochondrial matrix harbors the electron transport chain. In contrast, the outer membrane is highly permeable and permits low-molecular-weight molecules to move between the cytosol and the intermembrane gap [6,14]. A voltage-dependent anion channel, also known as a VDAC, may be found in the outer mitochondrial membrane. This channel acts as the principal transport pathway for ions and metabolites that are travelling across the membrane [15,16]. VDACs are responsible for a variety of essential cellular processes, such as the regulation of apoptosis signaling, the maintenance of synaptic plasticity through the mitochondrial permeability transition pore, and the regulation of the shape and structure of mitochondria. VDACs also play a role in maintaining synaptic plasticity through the mitochondrial permeability transition pore [16].

A recent study conducted in our group found that phosphorylated TAU interacts with VDAC1 in postmortem AD brains, as well as in the brains of APP transgenic animal models. These interactions became more prevalent as the disease advanced, which lends credence to the hypothesis that phosphorylated TAU, may obstruct the transport of organelles between mitochondria and the cytoplasm, potentially leading to flaws in oxidative phosphorylation and the production of mitochondrial ATP [16,17,18]. Further, in a recent study of VDAC1^+/−^/TAU mice, we showed that a partial decrease in VDAC1 reversed the TAU-induced behavioral deficits in VDAC1^+/−^/TAU mice compared to TAU animals. These behavioral impairments included motor coordination and exploratory behavioral alterations, as well as learning and spatial memory impairments [19]. In comparison to TAU animals, the protein levels of synaptic proteins, mitophagy proteins, and autophagy proteins were dramatically elevated in double-mutant mice. In addition to this, the number of dendritic spines was greatly increased, the number of mitochondria was significantly decreased, and the length of the mitochondria was significantly raised in double-mutant mice [19]. However, it is unclear whether a partial reduction in VDAC1 expression reduces mitochondrial fission, and increases fusion and biogenesis activities. To address these questions, in this present study, we studied 1) mitochondrial fission (DRP1, FIS1) and fusion (MFN1, MFN2, OPA1) proteins, and 2) mitochondrial biogenesis (PGC1A, NRF1, NRF2, TFAM) proteins using 6-month-old WT, VDAC1^+/−^, TAU, and VDAC1^+/−^/TAU mice.

## 2. Results

### 2.1. VDAC1^+/−^ Heterozygous Knockout Mice Decrease Mitochondrial Fission and Increase Mitochondrial Fusion Protein Expression

**Figure 1:**
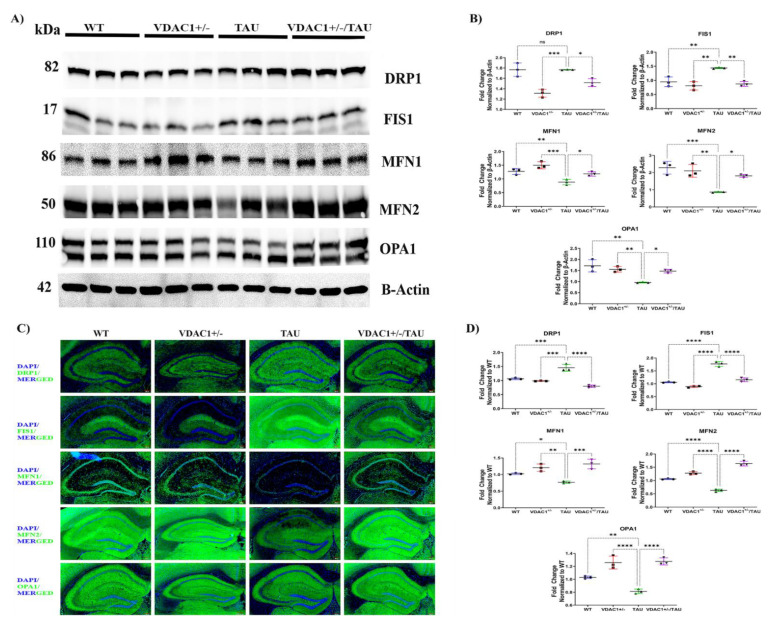
Western blot, immunofluorescence, and quantification analysis of proteins regulating mitochondrial dynamics in 6-month-old WT, VDAC1^+/−^, TAU, and VDAC1^+/−^/TAU mice. (**A**) Representative immunoblots. (**B**) Quantitative densitometry study of mitochondrial dynamics found that the fission of DRP1 (* *p* < 0.05) and FIS1 (** *p* < 0.01) was dramatically decreased, whereas the fusion of MFN1, MFN2, and OPA1 (* *p* < 0.05) was significantly enhanced in VDAC1^+/−^/TAU animals compared to TAU mice. Forty micrograms (g) of total protein were put into each lane. The loading control was carried out with the help of the housekeeping protein beta-actin. Data are from three independent experiments showed similar results (N = 3). Three animals were randomly selected from each group/genotype for immunoblotting and immunofluorescence analyses from a total of ten animals studied for behavioral phenotype [19]. (**C**) Representative immunofluorescence images of 10-micron coronal sections (10×). (**D**) Fluorescence intensity analysis of mitochondrial dynamics-DRP1 (**** *p* < 0.0001), FIS1 (**** *p* < 0.0001) (fission) was significantly decreased, MFN1 (*** *p* < 0.001), MFN2 (**** *p* < 0.0001) and OPA1 (**** *p* < 0.0001) (fusion) were significantly increased in VDAC1^+/−^/TAU mice compared to TAU mice. The data are from three separate experiments, all of which yielded comparable findings (N = 3), and each mouse was exposed to 10–15 fields. Scale bar: 200 μm. The results were presented as the mean accompanied by the standard error of the mean; ns denotes that the difference did not reach statistical significance; * *p* < 0.05, ** *p* < 0.01, *** *p* < 0.001, **** *p* < 0.0001; one-way ANOVA followed by Turkey’s test for multiple comparisons.

We characterized whether and how mitochondrial dynamics (fission and fusion) are affected in VDAC1^+/−^ mice. To examine changes in the expression of proteins involved in mitochondrial fission and fusion, we performed Western blot (using cerebral cortical tissues) and immunofluorescence (using hippocampal sections) analyses from the WT, VDAC1^+/−^, TAU, and VDAC1^+/−^/TAU mice. When compared to WT mice, the levels of the fission proteins DRP1 and FIS1 were significantly elevated in TAU animals. On the other hand, compared to WT mice, the levels of the mitochondrial fusion proteins MFN1, MFN2, and OPA1 were much lower in the TAU animals (Figure 1A,B). In a surprising finding, the mitochondrial fission proteins DRP1 and FIS1 were shown to be lower in VDAC1^+/−^ and VDAC1^+/−^/TAU mice in comparison to TAU animals. In contrast, mitochondrial fusion proteins MFN1, MFN2, and OPA1 were dramatically enhanced in the VDAC1^+/−^ and VDAC1^+/−^/TAU mice compared to TAU animals. The same trend was observed in the immunofluorescence analysis in hippocampal sections (Figure 1C,D) with, briefly, increased levels of mitochondrial fusion and decreased fission proteins in VDAC1^+/−^ and VDAC1^+/−^/TAU mice compared to TAU mice (Figure 1A–D, Figure S1).

### 2.2. Reduced Expression of VDAC1 Induces Mitochondrial Biogenesis in VDAC1^+/−^/TAU Mice

**Figure 2:**
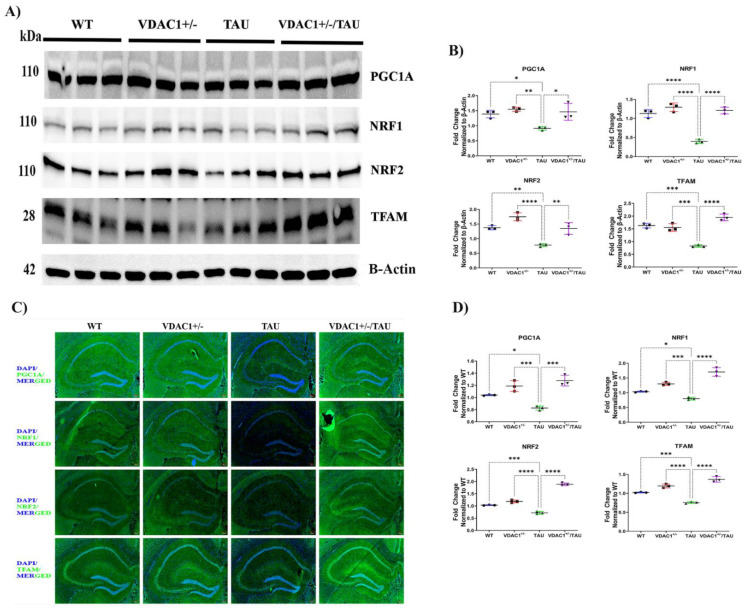
Western Blot, immunofluorescence, and quantification analysis of mitochondrial biogenesis proteins in the hippocampal fields of 6-month-old WT, VDAC1^+/−^, TAU, and VDAC1^+/−^/TAU mice. (**A**) Representative immunoblots. (**B**) Quantitative densitometry analysis of mitochondrial biogenesis PGC1A (* *p* < 0.05), NRF1 (**** *p* < 0.0001), NRF2 (** *p* < 0.01), and TFAM (**** *p* < 0.0001) proteins were significantly increased in VDAC1^+/−^/TAU mice compared to TAU mice. Forty micrograms (g) of total protein were put into each lane. The loading control was carried out with the help of the housekeeping protein beta-actin. Data are from three independent experiments with similar results (N = 3). (**C**) Representative immunofluorescence images of 10-micron coronal sections (10×). (**D**) fluorescence intensity analysis of mitochondrial biogenesis PGC1A (*** *p* < 0.001), NRF1 (**** *p* < 0.0001), NRF2 (**** *p* < 0.0001), and TFAM (**** *p* < 0.0001) proteins were significantly increased in VDAC1^+/−^/TAU mice compared to TAU mice. The data are from three separate experiments, all of which yielded comparable findings (N = 3), and each mouse was exposed to 10–15 fields. Scale bar: 200 μm. The results were presented as the mean accompanied by the standard error of the mean; ns denotes that the difference did not reach statistical significance; * *p* < 0.05, ** *p* < 0.01, *** *p* < 0.001, **** *p* < 0.0001; one-way ANOVA followed by Turkey’s test for multiple comparisons.

As shown in Figure 2A,B, significantly reduced levels of biogenesis proteins, PGC1A, NRF1, NRF2, and TFAM, were found in TAU mice compared to WT mice. On the contrary, mitochondrial biogenesis proteins, PGC1A, NRF1, NRF2, and TFAM, were significantly increased in VDAC1^+/−^ and VDAC1^+/−^/TAU mice compared to TAU mice. The same trend was observed in the immunofluorescence analysis of hippocampal sections (Figure 2C,D). To summarize, higher quantities of mitochondrial biogenesis proteins were found in VDAC1^+/−^ and VDAC1^+/−^/TAU animals in comparison to TAU mice (Figure 2A–D, Figure S2).

These results suggested that mitochondrial fusion and biogenesis were decreased, and mitochondrial fission was increased in TAU mice. However, these trends were reversed in the VDAC1^+/−^ and VDAC1^+/−^/TAU mice, suggesting that reduced expression of VDAC1 reduces the fission activity, and increases the fusion and biogenesis activities.

## 3. Discussion

AD is associated with mitochondrial dysfunction, which is a frequent clinical characteristic that leads to neurodegeneration [20,21,22]. Recent research has revealed that mitochondrial fission/fusion, and biogenesis are all changed in AD postmortem brains, as well as in both in vitro and in vivo models of AD [23,24,25]. We and other researchers have previously documented that P-TAU interacts with several mitochondrial proteins (DRP1 and VDAC1), which results in mitochondrial malfunction [15,17,18]. Our research group has previously reported that decreased levels of VDAC1 may result in decreased interaction between VDAC1 and P-TAU, which may ultimately result in normal mitochondrial function in AD [15]. In addition, we discovered that inhibiting the human VDAC1 gene in an in vitro environment might potentially increase synaptic activity, as well as mitochondrial maintenance and function, and provide protection against the harmful effects of AD-related genes [17]. In the present investigation, we have shown in vivo evidence that a partial decrease in VDAC1 mediated mitochondrial dynamics and biogenesis in a mouse model.

In mutant TAU mice, we examined the protective effects of a partial decrease in VDAC1 against the TAU-induced mitochondrial dysfunction. Through the process of breeding mutant TAU mice with VDAC1 heterozygote knockout (VDAC1^+/−^) mice, we were able to generate double-mutant animals [19]. In the preliminary investigation, using immunoblotting and immunofluorescence analysis, we measured the protein levels of mitochondrial dynamics and mitochondrial biogenesis in 6-month-old WT, VDAC1^+/−^, TAU, and VDAC1^+/−^/TAU mice. The dysfunction of mitochondria is linked to the course of illness in neurodegenerative disorders, and it is hypothesized that this dysfunction contributes to the excessive neuronal death seen in AD [26]. Fission (DRP1, FIS1) and fusion (MFN1, MFN2, OPA1) proteins are responsible for controlling the morphology of the mitochondria [10,27]. Our comparison of TAU mice and double-mutant (VDAC1^+/−^/TAU) mice revealed that reduced expression of fission proteins and increased levels of fusion proteins, as well as increased levels of mitochondrial biogenesis proteins, in double-mutant mice, indicate that reduced VDAC1 is protective against mutant TAU-induced mitochondrial toxicity. These findings were gleaned from a study in which we compared protein data from TAU mice and double-mutant mice.

In summary, the findings of the current study, together with our earlier studies of synaptic, mitophagy, autophagy, transmission electron microscopy, and behavioral phenotype analysis [19] provided evidence of the protective effects of reduced VDAC1 against the mitochondrial and synaptic toxicities induced by P-TAU in TAU (P301L) mice. These findings also offered new evidence to support the development of VDAC1 therapeutic strategies for AD.

## 4. Materials and Methods

### 4.1. Animals

We used VDAC1^+/−^ heterozygote knockout mice and mutant TAU mice (P301L line), in order to investigate the partial decrease in VDAC1. Previous research [28] has provided a description of how VDAC1^+/−^ mice are produced [18]. Mice carrying the human TAU P301L mutation [29] were used to produce the TAU strain, which were acquired from Taconic Biosciences (Germantown, NY, USA). The VDAC1^+/−^ mice were genetically crossed with the TAU mice, which resulted in the creation of the double-mutant mice known as VDAC1^+/−^/TAU. We genotyped the VDAC1^+/−^ and TAU mutations by utilizing DNA that was produced from a tail biopsy and performing PCR amplification, as was reported before [28,29]. Mice were bred and housed in the Laboratory Animal Resource Center at Texas Tech University Health Sciences Center, which is accredited by the Association for Assessment and Accreditation of Laboratory Animal Care International (AAALAC, #000989). The standard light–dark cycle for mice was 12 h long, and the lights were turned on at 7 a.m. each morning. The Institutional Animal Care and Use Committee at the Texas Tech University Health Sciences Center gave their stamp of approval to each and every experimental protocol (TTUHSC-IACUC, #16007).

### 4.2. Immunoblotting

Immunoblotting analysis was carried out as described earlier [30]. Briefly, Sonication in a RIPA buffer (Thermo Scientific, Waltham, MA, USA, Catalog number: 89901) with Halt^TM^ Protease and Phosphatase Inhibitor (Thermo Scientific: 78444) and EDTA was used to homogenize tissues from the cerebral cortex. The protein lysates were first placed on ice for a 20-min incubation period, with the tubes being occasionally stirred. Additional lysates were cleaned up by centrifuging them at 4 degrees Celsius for twenty minutes at 13,000× *g*. The BCA protein assay was used to determine the levels of protein in the samples (Thermo Scientific: 23222). Electrophoresis was performed using Mini-PROTEAN^®^ TGX Precast Protein Gels (10%, 12%, and 4–20%, Bio-Rad Laboratories, Hercules, CA, USA) after equal quantities of protein were diluted in a 4X Bolt^TM^ LDS Sample Buffer (Thermo Scientific: B0007). After that, the proteins were transferred using the Trans-Blot Turbo Transfer System onto PVDF membranes (BIO-RAD, Catalog number: 10026933). After one hour, the membranes were blocked with either 5% bovine serum albumin (BSA) or 5% non-fat skimmed milk. The processes of mitochondrial fission proteins: DRP1 (12957-1-AP, Rabbit Polyclonal 1:1000; Protein Tech Group, Rosemont, IL, USA), FIS1 (NB100-56646, Rabbit Polyclonal 1:500; Novus Biologicals, Littleton, CO, USA), fusion proteins: MFN1: (13798-1-AP, Rabbit Polyclonal 1:500; Protein Tech Group), MFN2 (9482, Rabbit Polyclonal 1:1000; Cell Signaling Technology, Danvers, MA, USA), OPA1 (NBP2-59770, Rabbit Polyclonal 1:1000; Novus Biologicals) and biogenesis proteins: PGC1A (NBP1-04676, Rabbit Polyclonal 1:1000; Novus Biologicals), NRF1 (46743, Rabbit Polyclonal 1:1000; Cell Signaling Technology), NRF2 (NBP1-32822, Rabbit Polyclonal 1:1000; Novus Biologicals), TFAM (ab131607, Rabbit Polyclonal 1:2000; Abcam, Waltham, MA, USA) were investigated using immunoblotting. After washing the membranes with a TBST buffer three times, for ten minutes at a time, they were subjected to an incubation period of one hour with the appropriate secondary antibodies, which was then followed by three further washes for ten minutes at a time. Chemiluminescent detection was utilized using the ImageQuant LAS-4000 (GE Healthcare Life Sciences, Chicago, IL, USA) and ECL (Thermo Scientific: 34076) in order to analyze the blots. Using the ImageJ program (Wayne Rasband and contributors National Institute of Health, Bethesda, MD, USA), the amount of intensity of each of the different protein bands was measured [30,31].

### 4.3. Immunofluorescence

Following the procedures outlined in the prior article [32], brain slices from the mice were prepared for immunofluorescence. After fixing the sections for ten minutes in a solution of one percent paraformaldehyde in PBS, the sections were rinsed three times in TBST. After that, the sections were blocked at room temperature for one hour using SuperBlock^TM^ Blocking Buffer (Thermo Scientific: 37515). After that, the sections were incubated with the appropriate primary antibodies [DRP1 (NB110-55288, Rabbit Polyclonal 1:100; Novus Biologicals), FIS1 (NB100-56646, Rabbit Polyclonal 1:100; Novus Biologicals), fusion proteins: MFN1: (13798-1-AP, Rabbit Polyclonal 1:100; Protein Tech Group), MFN2 (9482, Rabbit Polyclonal 1:100; Cell Signaling Technology), OPA1 (NBP2-59770, Rabbit Polyclonal 1:100; Novus Biologicals), PGC1A (NBP1-04676, Rabbit Polyclonal 1:100; Novus Biologicals), NRF1 (ab34682, Rabbit Polyclonal 1:100; Abcam), NRF2 (NBP1-32822, Rabbit Polyclonal 1:100; Novus Biologicals), TFAM (NBP2-19437, Rabbit Polyclonal 1:100; Novus Biologicals)] for a whole night at 4 degrees Celsius. Following incubation, the tissues were subsequently rinsed with TBST. After that, the tissues were prepared for incubation at room temperature for one hour with a secondary antibody that was conjugated with Alexa Fluor 488-goat-anti-rabbit (Invitrogen, Waltham, MA, USA, Catalog number: A32731) and Alexa Flour 594-goat-anti-mouse (Invitrogen: A11005). The slides were stained with DAPI for 15 min as a part of the nuclear staining process before being covered with anti-fade mounting liquid and glass. Using an Olympus IX83 microscope, immunofluorescent slices were viewed, and photographs were recorded (Olympus, Bartlett, TN, USA). With the help of the ImageJ program (Wayne Rasband and contributors National Institute of Health, Bethesda, MD, USA), the quantification of the staining intensity as well as the background fluorescence intensity was accomplished [30,32].

### 4.4. Statistical Analysis

The data were shown as the mean together with the standard error of the mean (S.E.M.). Using the GraphPad^TM^ PRISM program (version 9.4; GraphPad Software, La Zolla, CA, USA), statistical analyses were performed, and conclusions were reached based on those findings. Tukey’s test for multiple comparisons was utilized throughout the one-way analysis of variance that was carried out. When the *p*-value was less than 0.05 (*p* < 0.05), we determined that the comparisons between the groups were significant.

## Data Availability

Not applicable.

## Supplementary Materials

The following are available online at https://www.mdpi.com/article/10.3390/ijms23158561/s1.
