# Supplementary material for: Reduced VDAC1, Maintained Mitochondrial Dynamics and Enhanced Mitochondrial Biogenesis in a Transgenic Tau Mouse Model of Alzheimer’s Disease
Source: Int J Mol Sci. 2022 Aug 2;23(15):8561. doi: 10.3390/ijms23158561 (PMC9368852; doi:10.3390/ijms23158561)
Supplement: Supplementary file 1 [file ijms-23-08561-s001.zip › ijms-1837926-supplementary.pdf]

Supplemental Figure S1

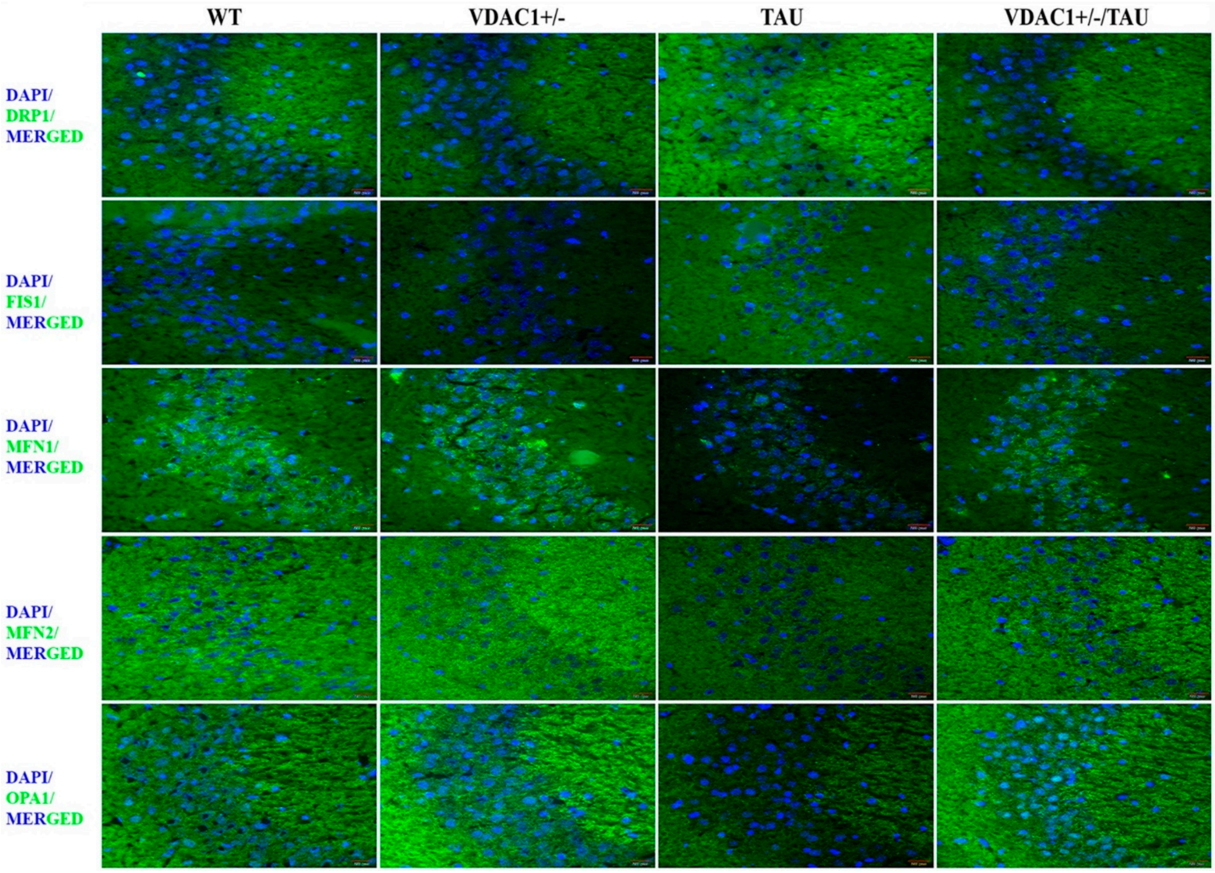

Supplemental Figure S1. Immunofluorescence images of mitochondrial dynamic proteins in the hippocampal fields of 6-months-old WT, VDAC1$^{+/-}$, TAU, and VDAC1$^{+/-}$/TAU mice. Representative Immunofluorescence images of 10 micron coronal sections (60X) of mitochondrial dynamics-DRP1, FIS1 (fission), MFN1, MFN2, OPA1 (fusion). The data are from three separate experiments, all of which yielded comparable findings (N = 3), and each mouse was exposed to 10–15 fields. Scale bar: 20 µm. The results were presented as the mean accompanied by the standard error of the mean. ns denotes that the difference did not reach statistical significance, *P < 0.05, **P < 0.01, ***P < 0.001, ****P < 0.0001, one-way ANOVA followed by Turkey’s test for multiple comparisons.

Supplemental Figure S2

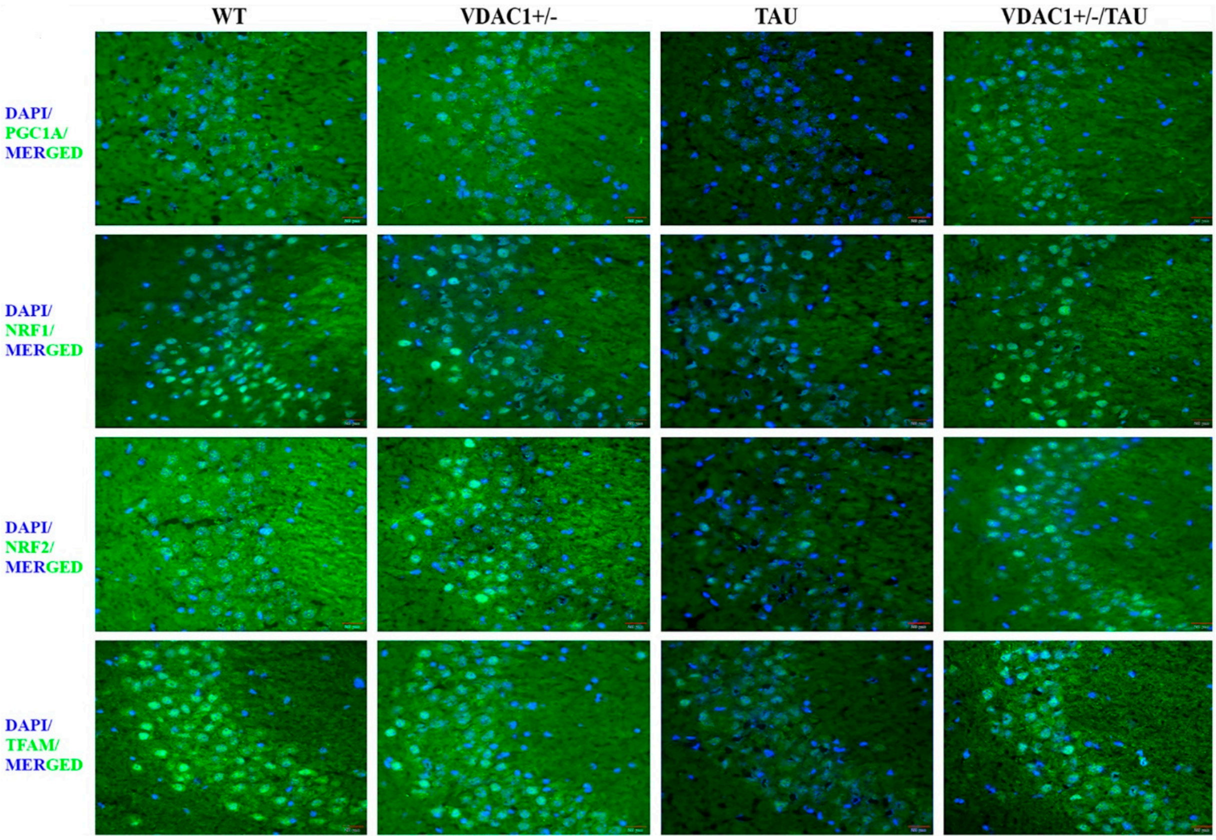

Supplemental Figure S2. Immunofluorescence images of mitochondrial biogenesis proteins in the hippocampal fields of 6-months-old WT, VDAC1$^{+/-}$, TAU, and VDAC1$^{+/-}$/TAU mice. Representative Immunofluorescence images of 10 micron coronal sections (60X) of mitochondrial biogenesis PGC1A, NRF1, NRF2 and TFAM proteins. The data are from three separate experiments, all of which yielded comparable findings (N = 3), and each mouse was exposed to 10–15 fields. Scale bar: 20 µm. The results were presented as the mean accompanied by the standard error of the mean. ns denotes that the difference did not reach statistical significance, *P < 0.05, **P < 0.01, ***P < 0.001, ****P < 0.0001, one-way ANOVA followed by Turkey's test for multiple comparisons.
